# Heterogeneous patterns of old-age vulnerability and loneliness from the cross-national comparative perspective

**DOI:** 10.1093/geroni/igab046.3696

**Published:** 2021-12-17

**Authors:** Oejin Shin, Sojung Park, Ji young Kang, Minyoung Kwak, Jihye Baek

**Affiliations:** 1 University of Illinois at Urbana Champaign, Savoy, Illinois, United States; 2 Washington university, St.Louis, Missouri, United States; 3 Chungnam National University, Daejeon, Ch'ungch'ong-namdo, Republic of Korea; 4 Daegu University, Gyeongsan, Gyeongbuk, Kyongsang-bukto, Republic of Korea; 5 Gwanak Senior Welfare Center, St. Louis, Missouri, United States

## Abstract

Loneliness has been extensively examined as a major risk factor for mortality and morbidity among older adults. In this research, we identified multi-dimensional patterns of old-age vulnerability in five countries (U.S., Korea, Sweden, Germany, Italy) guided by the welfare regime framework and examined how the patterns of vulnerability are associated with loneliness in old age in each country. Data were drawn from comparative national aging data: HRS for the U.S. (n=4331), KLOSA for Korea (n=3721), SHARE for Sweden (n=2746), Germany (n=2271), and Italy (n=2988). We used three vulnerability constructs: 1) material vulnerability (poverty, high out-of-pocket expenses for health care, unaffordability for housing), 2) health vulnerability (multiple chronic condition, depression), and 3) social vulnerability (living alone, contact with children and friend, social participation). Latent Class Analysis and regression analyses were used for data analysis. The relative proportion of the least vulnerable group varies substantially (from 11.88% in Italy to 59.33% in the U.S). In Sweden and Germany, around 7% of the sample belongs to the most vulnerable group, while in other countries the most vulnerable group was not found. Notably, in Italy, older people in the social vulnerable group were more likely to feel lonely when compared to least vulnerable, suggesting the country-specific significance of social supportive environment. This study is the first research to examine the empirical structure of the concept and how it may vary across countries related to their loneliness. A full discussion of country-specific discussion and policy implication will be presented.

